# Clinical significance of serum-derived exosomal PD-L1 expression in patients with advanced pancreatic cancer

**DOI:** 10.1186/s12885-023-10811-8

**Published:** 2023-05-01

**Authors:** Se Jun Park, Ju Yeon Park, Kabsoo Shin, Tae Ho Hong, MyungAh Lee, Younghoon Kim, In-Ho Kim

**Affiliations:** 1 Division of Medical Oncology, Department of Internal Medicine, The Catholic University of Korea, Seoul St. Mary’s Hospital, 222 Banpo-daero, Secho-gu, Seoul, Korea; 2 Cancer Research Institute, College of Medicine, The Catholic University of Korea, Seoul, Korea; 3 Department of General Surgery, The Catholic University of Korea, Seoul St. Mary’s Hospital, Seoul, Korea; 4 Department of Pathology, The Catholic University of Korea, Seoul St. Mary’s Hospital, College of Medicine, Seoul, Korea

**Keywords:** Pancreatic cancer, Exosomes, Programmed death ligand 1, Prognosis

## Abstract

**Background:**

Interactions between the programmed cell death receptor 1 (PD-1) and its ligand (PD-L1) lead to immune evasion in various tumors and are associated with poor prognosis in patients with pancreatic cancer; however, the roles of PD-L1-containing exosomes in pancreatic cancer is poorly understood. Here, we investigated the correlation between circulating exosomal PD-L1 (exoPD-L1) and PD-L1 expression in tumor tissue, and survival outcomes in patients with advanced PDAC.

**Methods:**

Exosomes were derived from pre-treatment serum samples isolated using ExoQuick kit from 77 patients with advanced pancreatic cancer. Exosomal PD-L1 (exoPD-L1) was detected by enzyme-linked immunosorbent assay, and matched tumor tissues PD-L1 expression were evaluated by PD-L1 immunohistochemistry (22C3) assay, described with combined positive score. Cutoff value of exoPD-L1 for survival was assessed with receiver operating characteristic curve analysis. Kaplan-Meier analysis was performed to obtain median overall survival (OS), and hazard ratio was estimated using a stratified Cox regression model.

**Results:**

The median exoPD-L1 serum concentration was 0.16 pg/mg, with undetected levels in seven patients. ExoPD-L1 levels were significantly higher in patients with systemic disease than in those with locally advanced disease (*p* = 0.023). There was a significantly higher proportion of elevated exoPD-L1 levels in patients with positive PD-L1 expression compared to patients with negative PD-L1 expression (*p* = 0.001). Patients were classified into groups with low and high exoPD-L1 levels using ROC curve-derived cutoffs (0.165 pg/mg; area under the curve, 0.617; *p* = 0.078). At a median follow-up of 8.39 months, the median OS was 13.2 (95% CI, 8.17–18.3) and 6.36 months (95% CI, 3.27–9.45) in the low and high exoPD-L1 groups, respectively (HR = 0.61; 95% CI, 0.35–1.04; *p* = 0.059). ExoPD-L1 levels did not affect the proportion of CD8^+^CD69^+^ effector cytotoxic T cells in either of the groups (*p* = 0.166).

**Conclusions:**

The serum-derived exoPD-L1 levels were higher in metastatic pancreatic cancer than locally advanced disease. Collectively, higher serum exoPD-L1 levels in patients with advanced pancreatic cancer suggested worse survival outcomes and may have clinical implications.

**Supplementary Information:**

The online version contains supplementary material available at 10.1186/s12885-023-10811-8.

## Introduction

Pancreatic ductal adenocarcinoma (PDAC) is a lethal disease with a dismal 5-year overall survival (OS) rate of only 9% [1]. Although therapeutic strategies have considerably improved, less than 20% patients with PDAC are eligible for curative resection at diagnosis [2]. Chemotherapeutic efficacy is unsatisfactory owing to the unique PDAC tumor microenvironment (TME) and immune evasion by tumor-infiltrating immunosuppressive cells [3]. Particularly, the advancement of targeted agents and immunotherapy for patients with PDAC has been limited [4].

In many solid tumors, T-cell activation is inhibited by the interaction between the immune checkpoint programmed cell death ligand 1 (PD-L1) and programmed cell death receptor 1 (PD-1), which is an inhibitory receptor of activated T-cells. Suppression of T cell activation facilitates immune evasion by cancer cells and is associated with poor prognosis in patients with cancer [5]. Thus, the PD-1/PD-L1 pathway is a promising target for cancer treatment. Immune checkpoint inhibitors (ICI) have demonstrated remarkable clinical success in several solid tumors, including non-small cell lung carcinoma [6]. However, clinical trials of a single ICI for patients with advanced PDAC have shown modest efficacy [7]

The TME of PDAC comprises regulatory T cells (Tregs), myeloid-derived suppressive cells (MDSCs), and tumor-associated macrophages (TAMs) that inhibit effector CD8 + T cells [8–10]. PD-L1 overexpression on the surface of tumor cells correlates with worse survival outcomes in patients with PDAC [11]. However, the prognostic value of tumor PD-L1 in PDAC remains controversial due to the use of non-standardized methods and heterogenous samples. Moreover, tumor cells secrete various forms of PD-L1 into the extracellular environment, including cleaved or secreted soluble PD-L1 (sPD-L1) and exosomal PD-L1 (exoPD-L1) [12]. Exosomes are extracellular vesicles that contain various bioactive molecules, such as non-coding and micro RNAs, mRNAs, signaling proteins, and cytokines, and mediate intercellular signal communication [13]. ExoPD-L1 can mediate immunosuppression locally or systemically by interacting with immune cells expressing PD-1. In addition, exoPD-L1 may exhibit a more potent immunosuppressive activity than other forms of extracellular PD-L1 [12]. ExoPD-L1 is associated with poor postoperative outcomes in patients with gastric cancer and is correlated with advanced stages in patients with head and neck cancer [14, 15]. In patients with metastatic melanoma, the level of circulating exoPD-L1 was associated with anti-PD-1 response [16]. Although circulating sPD-L1 was negatively correlated with survival in patients with metastatic PDAC, the clinical significance of exoPD-L1 in patients with PDAC has not been investigated [17]

In the present study, we investigated the correlation between PD-L1 expression in tumor tissue and circulating exoPD-L1 in patients with advanced PDAC. Furthermore, we evaluated whether circulating exoPD-L1 is associated with survival outcomes in patients with advanced PDAC. We also examined the correlation between CD8 + T cell counts and circulating exoPD-L1 levels.

## Materials and methods

### Patients and samples

We enrolled 77 patients with advanced PDAC diagnosed at the Catholic University of Korea, Seoul St. Mary’s Hospital, between 2017 and 2021. Patients with available serum samples at the time of diagnosis for advanced disease were eligible according to the following criteria: (1) histologically confirmed PDAC; (2) locally advanced or metastatic disease; (3) survival confirmed at the time of data collection; (4) Eastern Cooperative Oncology Group (ECOG) performance status (PS) from 0 to 2. Peripheral blood samples were collected from patients before initiating systemic chemotherapy in a first-line palliative setting. Blood samples were delivered to the laboratory, immediately centrifuged at 2,500 × g for 10 min and stored in 2–4 mL aliquots at − 80 °C until use. Tumor tissue specimens were obtained from patients with PDAC who had undergone surgery or core needle biopsy. The study protocol was approved by the Institutional Review Board (IRB) of The Catholic University of Korea, Seoul St. Mary’s Hospital (approval ID: KC19TESI0377). Informed consent was obtained from all the patients. All procedures were conducted in accordance with Korean regulations and the Declaration of Helsinki.

### Exosome isolation and identification from serum samples

Serum exosomes were isolated using the ExoQuick exosome precipitation solution (System Biosciences, Mountain View, CA, USA) as per manufacturer’s instructions. Thawed serum samples (500 µL) were centrifuged at 12,000 × g for 15 min to remove cells and cell debris. The supernatants were transferred to a sterile tube, mixed with ExoQuick reagent (120 µL) and incubated for 1 h at 4 °C, and then centrifuged at 1,500 × g for 30 min. The supernatant was removed, and the precipitate was centrifuged at 1,500 × g for 5 min to remove the residual ExoQuick solution and obtain precipitated exosome in the pellet. After discarding the supernatant, the pellets were resuspended in 250 µL sterile phosphate-buffered saline (PBS) and stored at − 80 °C until required. The NanoSight NS300 instrument with nanoparticle tracking analysis 2.3 software (Malvern Instruments Ltd, Malvern, UK) was used to analyze exosomal size distribution [18]. Exosomes were characterized using transmission electron microscopy (JEM1010, JEOL, Tokyo, Japan) at 80 kV [19]. To confirm the presence of exosomes, expression of exosome specific markers CD63 and TSG101 was evaluated using western blot analysis [20]

### Western blot analysis

Briefly, 50 µg exosomes were resolved by 12% SDS-PAGE and transferred to nitrocellulose membranes (Amersham Pharmacia, Sweden) for western blot analysis. The membranes were incubated with primary antibodies against TSG101 (#SC-7964, 1:100) and CD63 (#SC-5275, 1:200; Santa Cruz Biotechnology, CA, USA). Membranes were incubated with appropriate horseradish peroxidase-conjugated secondary antibodies (Bio-Rad, Hercules, CA, USA), and the bands were visualized using a chemiluminescent western blot reagent (Amersham Pharmacia, Sweden).

### Enzyme-linked immunosorbent assay (ELISA)

The expression of PD-L1 in serum-derived exosomes was detected using human PD-L1 DuoSet ELISA kits (#DY156, R&D Systems, Minneapolis, MN, USA) according to the manufacturer’s protocols. The optical density was measured at 450 nm using a VersaMax Microplate Reader (Molecular Devices, CA, USA), and protein levels were calculated according to standard curves. The protein concentration of the isolated exosomes was determined using a BCA protein assay kit (Thermo Scientific, Rockford, USA) according to the manufacturer’s instructions. The concentration of exoPD-L1 was standardized per mg of exosomal protein. For the detection of cytokines in the plasma, commercially available ELISA kits for transforming growth factor beta 1 (TGF-β1) (#DY240-05), TGF-β2 (#DY302), granzyme B (#DY2906-05), interferon-gamma (IFN-γ) (#DY285B-05), and interleukin (IL)-1β (#DY201-05, all from R&D Systems, Minneapolis, MN, USA) were used according to the manufacturer’s protocol.

### Defining a cohort with high serum exoPD-L1 levels

Higher levels of sPD-L1 are associated with a poor prognosis in patients with various solid tumors [21]. However, in patients with PDAC, the association between sPD-L1 expression and survival outcomes remains unknown as no data have been reported regarding the correlation between exoPD-L1 expression and prognosis [17, 22]. Therefore, there is no defined cutoff value of exoPD-L1 for prognosis in patients with PDAC. We used receiver operating characteristic (ROC) curve analysis to define an optimal prognostic cutoff value of exoPD-L1 in predicting survival nine months after enrollment, thereby classifying patients into two cohorts—high versus low exoPD-L1 levels—according to the ideal cutoff value.

### Evaluation of tumor PD-L1 expression

Formalin-fixed, paraffin-embedded tissues (4-µm-thick sections) from patients with advanced PDAC were tested for PD-L1 expression using the PD-L1 immunohistochemistry (IHC) 22C3 pharmDx assay (Dako, Carpinteria, CA, USA) on an Autostainer Link 48 system with an EnVision DAB Detection System (Agilent Technologies, Santa Clara, CA, USA) following the manufacturer’s instructions. PD-L1 immunoreactivity was evaluated by two pathologists using a multihead microscope. PD-L1 expression in the specimen was described using a combined positive score (CPS), which is defined as the number of PD-L1 stained cells (tumor cells, lymphocytes, and macrophages) divided by the total number of viable tumor cells and multiplied by 100 [23]

### Flow cytometry for detection of T cells

Peripheral blood mononuclear cells were isolated using Ficoll-Paque PLUS (#GE17-1440-02, Sigma-Aldrich, MA, USA) density-gradient centrifugation and stored in liquid nitrogen until further analysis. Thawed peripheral blood mononuclear cells were incubated with anti-CD3 (#317,314), anti-CD45 (#368,510), anti-CD8 (#300,910), and anti-CD69 (#310,912, all from BioLegend, CA, USA) antibodies for 20 min at 4 °C in the dark. The stained cells were analyzed using flow cytometry (BD FACSCanto II, BD Biosciences, CA, USA), and data were analyzed using FlowJo software (Tree Star, Ashland, OR, USA).

### Statistical analysis

Descriptive statistics are reported as proportions and medians with ranges or interquartile ranges (IQR). A time-dependent ROC curve was used to assess the predictive performance of the clinical factors associated with survival outcomes. The cutoff values of the clinical factors were calculated based on Youden’s index. Differences between groups and associations between clinicopathological features and exoPD-L1 were determined using chi-squared and Fisher’s exact tests for categorical data and an unpaired t-test with Welch’s correction for continuous data. Pearson’s correlation coefficient was used to evaluate the correlation between exoPD-L1 and tumor PD-L1 expression. OS was estimated from the date of diagnosis of advanced disease to the time of last follow-up or death. Survival curves were estimated using the Kaplan–Meier method, and survival differences were compared using a two-tailed log-rank test. Cox proportional hazards regression models were used to identify the effects of the clinical factors on survival. The hazard ratio (HR) and 95% confidence intervals (CIs) were estimated for each factor. Results with two-sided p-values < 0.05 were considered statistically significant. Statistical analyses were performed using SPSS for Windows (version 24.0; IBM SPSS Inc., Armonk, New York, USA) and GraphPad Prism version 8.0 (GraphPad Software Inc., San Diego, CA, USA).

## Results

### Clinicopathological characteristics of the study cohort

The clinicopathologic characteristics of the 77 patients with advanced PDAC are summarized in Table 1. The median age was 67 years, and most patients (80.5%) had an ECOG score of 0–1. Thirty-one patients (40.2%) had primary tumors in the pancreatic head, and 46 patients (59.8%) had tumors in the body and tail. Most patients (89.6%) had systemic disease, and eight (10.4%) had locally advanced disease without distant metastasis. Sixty-two patients (80.5%) were in an advanced stage, and 15 patients (19.5%) had recurrent disease. More than half of the patients (54.5%) had well or moderately differentiated cancer, and the majority (84.1%) had metastatic sites in less than two organs. Serum carbohydrate antigen 19 − 9 (CA 19 − 9) levels were > 59-fold higher than the upper limit of the normal range in 54 patients (70.1%) before palliative treatment.

**Table 1:** Clinicopathological characteristics and serum exoPD-L1 level of patients with pancreatic ductal adenocarcinoma

Variables	Total (n = 77)	ExoPD-L1 (pg/mg, median, IQR)	*p* value
**Age**, Median (Range)	67 (39–86)		
< 65 year, n (%) ≥ 65 year, n (%)	28 (36.4) 49 (63.6)	0.15 (0.05–0.18) 0.17 (0.14–0.20)	0.235
**Gender, n (%)**			
Male Female	44 (57.1) 33 (42.9)	0.16 (0.11–0.19) 0.16 (0.12–0.20)	0.319
**ECOG performance status, n (%)**			
0–1 2	62 (80.5) 15 (19.5)	0.16 (0.11–0.19) 0.20 (0.14–0.21)	0.269
**Tumor location, n (%)**			
Head Body/Tail	31 (40.2) 46 (59.8)	0.15 (0.11–0.19) 0.16 (0.14–0.20)	0.561
**Disease status, n (%)**			
Locally advanced Metastatic	8 (10.4) 69 (89.6)	0.08 (0-0.18) 0.16 (0.13–0.20)	**0.023**
**Previous tumor resection, n (%)**			
No (Initially advanced) Yes (Recurrent disease)	62 (80.5) 15 (19.5)	0.16 (0.13–0.19) 0.16 (0.11–0.20)	0.404
**Histologic grading, n (%)**			
Grade 1/2 Grade 3 Not available	42 (54.5) 17 (22.1) 18 (23.4)	0.16 (0.12–0.21) 0.14 (0.11–0.16)	0.465
Number of metastatic organ sites*			
1–2 ≥ 3	58 (84.1) 11 (15.9)	0.16 (0.13–0.20) 0.16 (0.14–0.18)	0.234
**Baseline CA19-9 level, n (%)**			
< 59×ULN (U/mL) ≥ 59×ULN (U/mL) Unknown	54 (70.1) 22 (28.6) 1 (1.3)	0.16 (0.11–0.19) 0.18 (0.14–0.22)	0.817
**Baseline albumin, n (%)**			
≥ 3.5 g/dL < 3.5 g/dL	53 (68.8) 24 (31.2)	0.15 (0.11–0.18) 0.18 (0.15–0.21)	0.243
Site of metastatic disease*, n (%)			
Liver Lung Peritoneum	47 (68.1) 14 (20.3) 21 (11.6)	0.16 (0.12–0.19) 0.17 (0.15–0.20) 0.17 (0.14–0.21)	0.390 0.470 0.289

*ExoPD-L1* exosomal programmed cell death ligand 1, *IQR* interquartile range, *ECOG* Eastern Cooperative Oncology Group, *CA 19 − 9* carbohydrate antigen 19 − 9, *ULN* the upper limit of the normal range. The normal range is 0–35 U/mL. *In population with metastatic disease

### ExoPD-L1 levels in patients with advanced pancreatic cancer

**Fig. 1:**
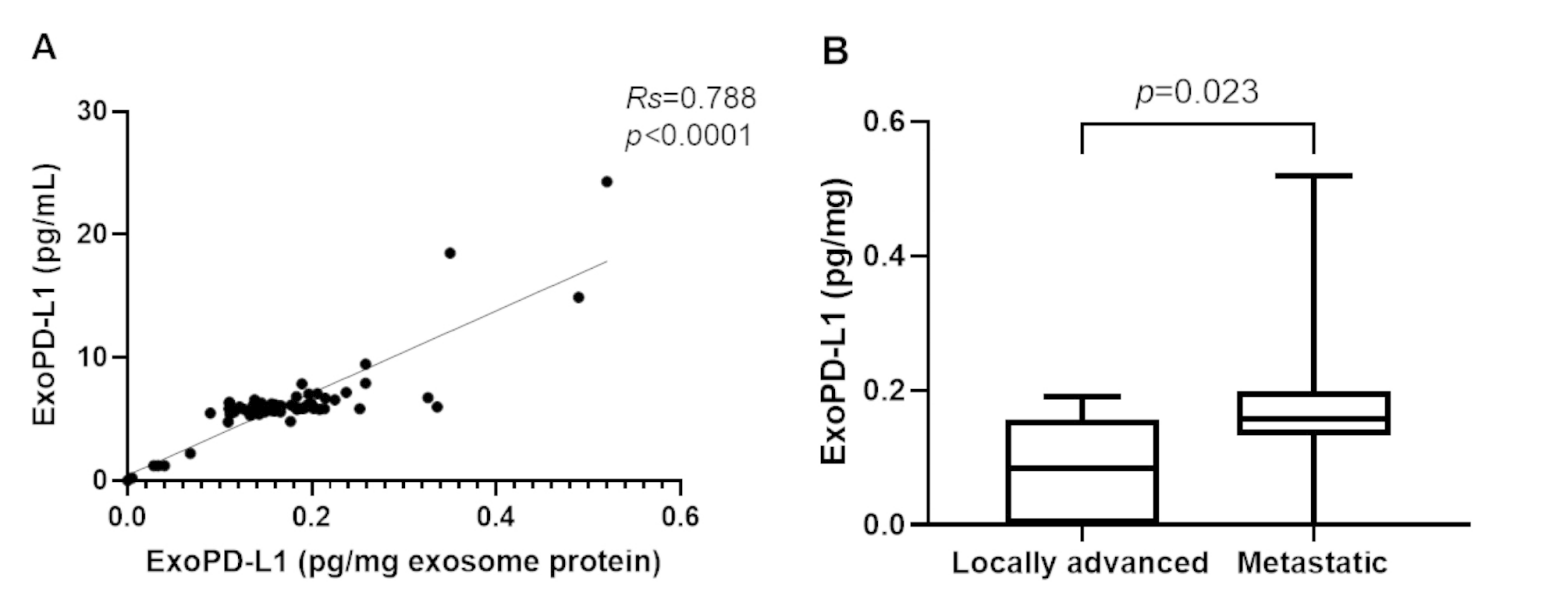
ExoPD-L1 profile and association with disease stage. (**A**) Correlation between serum and relative exoPD-L1 concentrations. (**B**) Association between disease status and serum exoPD-L1 levels

ExoPD-L1 was undetectable or below the lower detection limit in seven of the 77 patients (9.0%). The median value of exoPD-L1 was 5.87 (IQR, 5.50–6.27 pg/mL), and the median value of exoPD-L1 normalized with exosomal protein was 0.16 (IQR, 0.12–0.20 pg/mg). Standardized exoPD-L1 concentrations were associated with corresponding exoPD-L1 levels (*Rs* = 0.788; *p* < 0.0001; Fig. 1A, S1A). Patients with the systemic disease had significantly higher exoPD-L1 levels than those with locally advanced disease (*p* = 0.023; Table 1; Fig. 1B). In contrast, the serum levels of exoPD-L1 did not correlate with any other clinicopathological parameters (Table 1).

### Correlation between tissue PD-L1 and exoPD-L1 levels in patients with PDAC

**Fig. 2:**
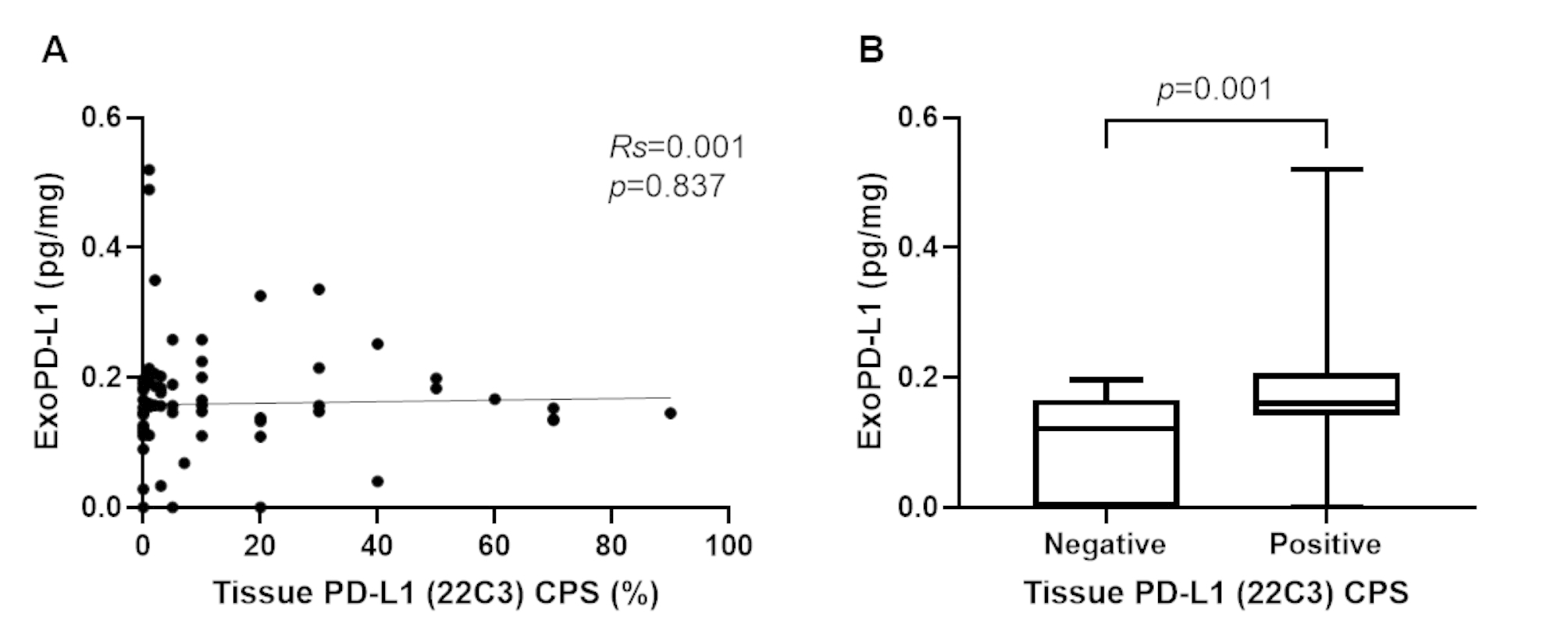
Relationship between tumor PD-L1 expression and serum exoPD-L1 levels. (**A**) No linear correlation was observed between tissue PD-L1 CPS score and exoPD-L1 levels. (**B**) ExoPD-L1 levels were higher in patients with positive tissue PD-L1 expression compared to those with negative tissue PD-L1 expression

Among the 77 patients, PD-L1 expression in the tumor tissue was evaluated in 68 patients, and the correlation between tissue PD-L1 and serum exoPD-L1 levels was assessed. The median CPS of tumor PD-L1 was 3.0 (IQR, 0–20), and 19 patients (27.9%) had negative tumor PD-L1 expression, defined as CPS < 1% (Figure S2). No linear correlation was observed between the tissue PD-L1 CPS and serum exoPD-L1 values in patients with advanced PDAC (*Rs* = 0.001; 95% CI, − 0.21–0.26; *p* = 0.837; Fig. 2A, S1B). However, as shown in Fig. 2B, a significantly higher proportion of elevated exoPD-L1 levels was observed in patients with positive tissue PD-L1 expression than in those negative for PD-L1 expression (*p* = 0.001).

### ExoPD-L1 levels and survival outcomes

**Fig. 3:**
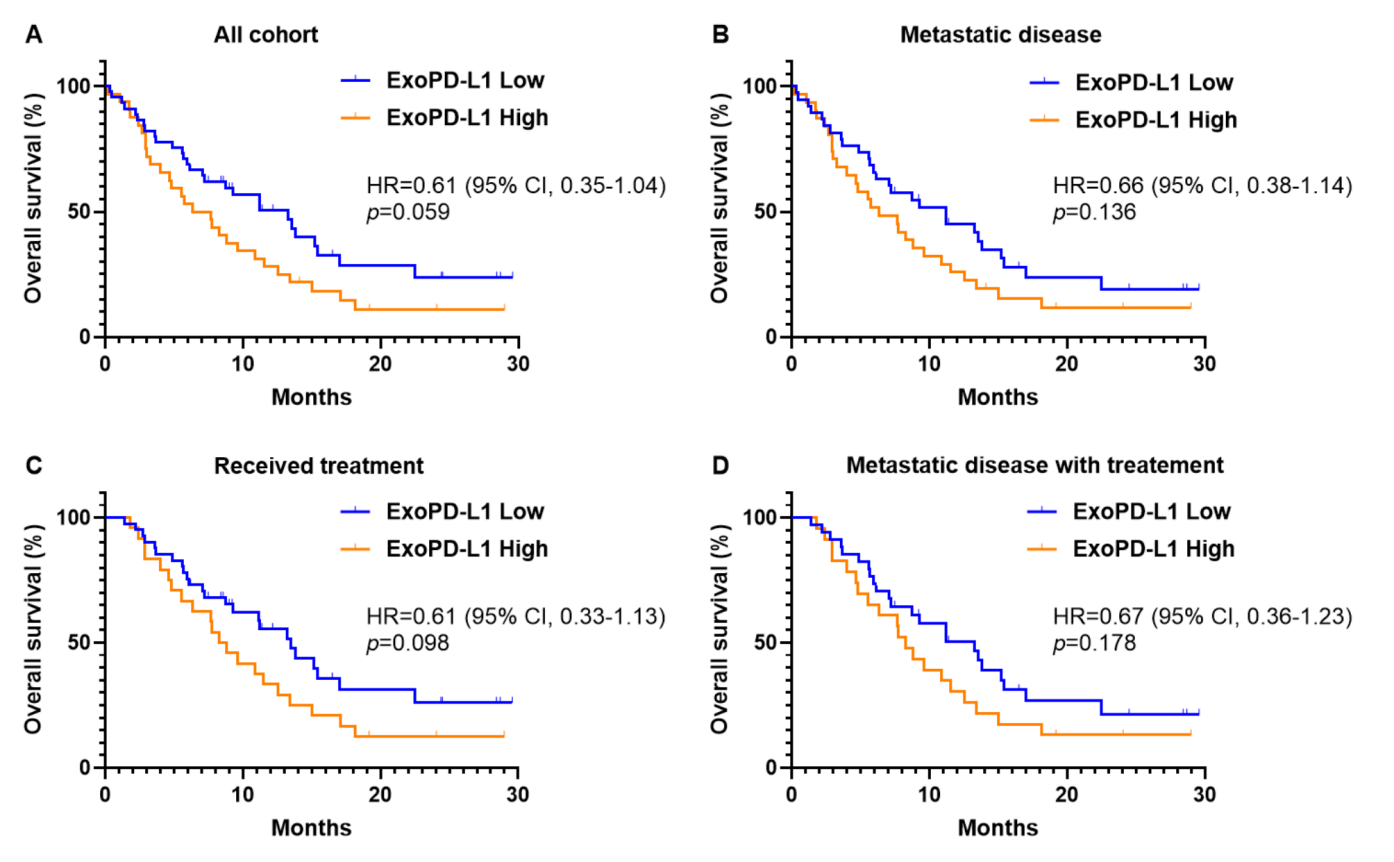
Kaplan–Meier estimates of overall survival according to serum exoPD-L1 levels. (**A**) Overall survival in all cohorts (n = 77), (**B**) in patients with metastatic disease (n = 69), (**C**) in patients who received systemic treatment (n = 65), and (**D**) in patients with metastatic disease who received chemotherapy (n = 57)

By the time of the final analysis (September 30, 2022), 56 patients (72.7%) had died. Among these deaths, 51 (92.7%) were related to disease progression, and five (8.9%) were attributed to treatment-related adverse events. The median follow-up was 8.39 months (95% CI, 6.10–10.9). In the entire cohort, the median OS was 9.28 months (95% CI, 5.91–12.6), and OS rates at 6 months, 9 months, and 1 year were 62.3%, 50.1%, and 40.6%, respectively. The optimal cutoff value that discriminated survivors at nine months was determined using the ROC curve. The optimal cutoff value of exoPD-L1 was 0.165 pg/mg, and the area under the curve was 0.617 (95% CI, 0.49–0.74, *p* = 0.078, Figure S3) with 52.6% sensitivity and 69.2% specificity. According to the cutoff value, the entire cohort was classified into low exoPD-L1 (45 patients) and high exoPD-L1 (32 patients) groups. The median OS was 13.2 months (95% CI, 8.17–18.3) in the low exoPD-L1 group, as compared with 6.36 months (95% CI, 3.27–9.45) in the high exoPD-L1 group (HR = 0.61; 95% CI, 0.35–1.04; *p* = 0.059; Fig. 3A). In 69 patients with metastatic disease, excluding locally advanced disease, the median OS was 11.2 months (95% CI, 4.97–17.4) and 6.36 months (95% CI, 3.14–9.58) in the low and high exoPD-L1 groups, respectively (HR = 0.66; 95% CI, 0.38–1.14; *p* = 0.136; Fig. 3B). In the group receiving systemic treatment (65 patients), the median survival was 13.5 months (95% CI, 9.78–17.2) in patients with low exoPD-L1 levels compared with 8.29 months (95% CI, 5.97–10.6) in patients with high exoPD-L1 levels (HR = 0.61; 95% CI, 0.33–1.13; *p* = 0.098; Fig. 3C). No significant differences were observed in median progression-free survival and response rate between the two groups based on exoPD-L1 levels among patients who received palliative systemic treatment (HR = 0.78; 95% CI, 0.43–1.43, *p* = 0.425, Figure S4, Table S1). In patients with metastatic disease who received systemic treatment (57 patients), the median OS was 13.2 months (95% CI, 8.24–18.2) in the low exoPD-L1 group and 8.29 months (95% CI, 6.55–10.0) in the high exoPD-L1 group (HR = 0.67; 95% CI, 0.36–1.23; *p* = 0.178; Fig. 3D), the difference of median OS between groups was not significant.

### Multivariate analysis for OS

The results of the univariate and multivariate analyses for OS are shown in Table 2, with subgroups based on the clinical parameters and exoPD-L1 levels. High exoPD-L1 levels tended to be associated with higher mortality in the univariate analysis; however, no relationship was observed between exoPD-L1 levels and OS in the multivariate analysis (HR = 1.18; 95% CI, 0.65–2.15; *p* = 0.579; Table 2). In the multivariate analysis, poor ECOG PS (ECOG, 2 vs. 0–1; HR = 2.22; 95% CI, 1.11–4.45; *p* = 0.024) and higher baseline neutrophil-to-lymphocyte ratio (NLR) (NLR, ≥ 3.5 vs. < 3.5; HR = 4.52; 95% CI, 2.46–8.30; *p* < 0.001) were significantly associated with worse OS. In addition, patients who received systemic chemotherapy had longer OS (HR = 0.31; 95% CI, 0.14–0.71; *p* = 0.005) than those who did not (Table 2).

**Table 2:** Univariate and multivariate analyses of the clinical features and exoPD-L1 levels for overall survival in patients with pancreatic cancer

	Overall survival			
Variables	Univariate analysis		Multivariate analysis	
	HR (95% CI)	*p* value	HR (95% CI)	*p* value
Age ≥ 65 (vs. <65 year)	1.96 (1.10–3.51)	0.024	1.78 (0.90–3.51)	0.097
ECOG PS 2 (vs. ECOG PS 0–1)	2.97 (1.58–5.58)	0.001	2.22 (1.11–4.45)	0.024
Received chemotherapy (vs. none)	0.19 (0.09–0.37)	< 0.001	0.31 (0.14–0.71)	0.005
Metastatic disease (vs. locally advanced)	3.92 (0.95–16.1)	0.058	3.33 (0.77–14.4)	0.108
ExoPD-L1 high (vs. low)	1.65 (0.98–2.79)	0.062	1.18 (0.65–2.15)	0.579
NLR ≥ 3.5 (vs. <3.5)	3.17 (1.85–5.43)	< 0.001	4.52 (2.46–8.30)	< 0.001
CA 19 − 9 ≥ 59×ULN (vs. <59×ULN)	1.92 (1.09–3.35)	0.023	1.33 (0.72–2.46)	0.370

*ExoPD-L1* exosomal programmed cell death ligand 1, *HR* hazard ratio, *CI* confidence interval, *ECOG PS* Eastern Cooperative Oncology Group Performance Status, *NLR* neutrophil-to-lymphocyte ratio, *CA 19 − 9* carbohydrate antigen 19 − 9

### ExoPD-L1 and cytotoxic T cells in patients with PDAC

**Fig. 4:**
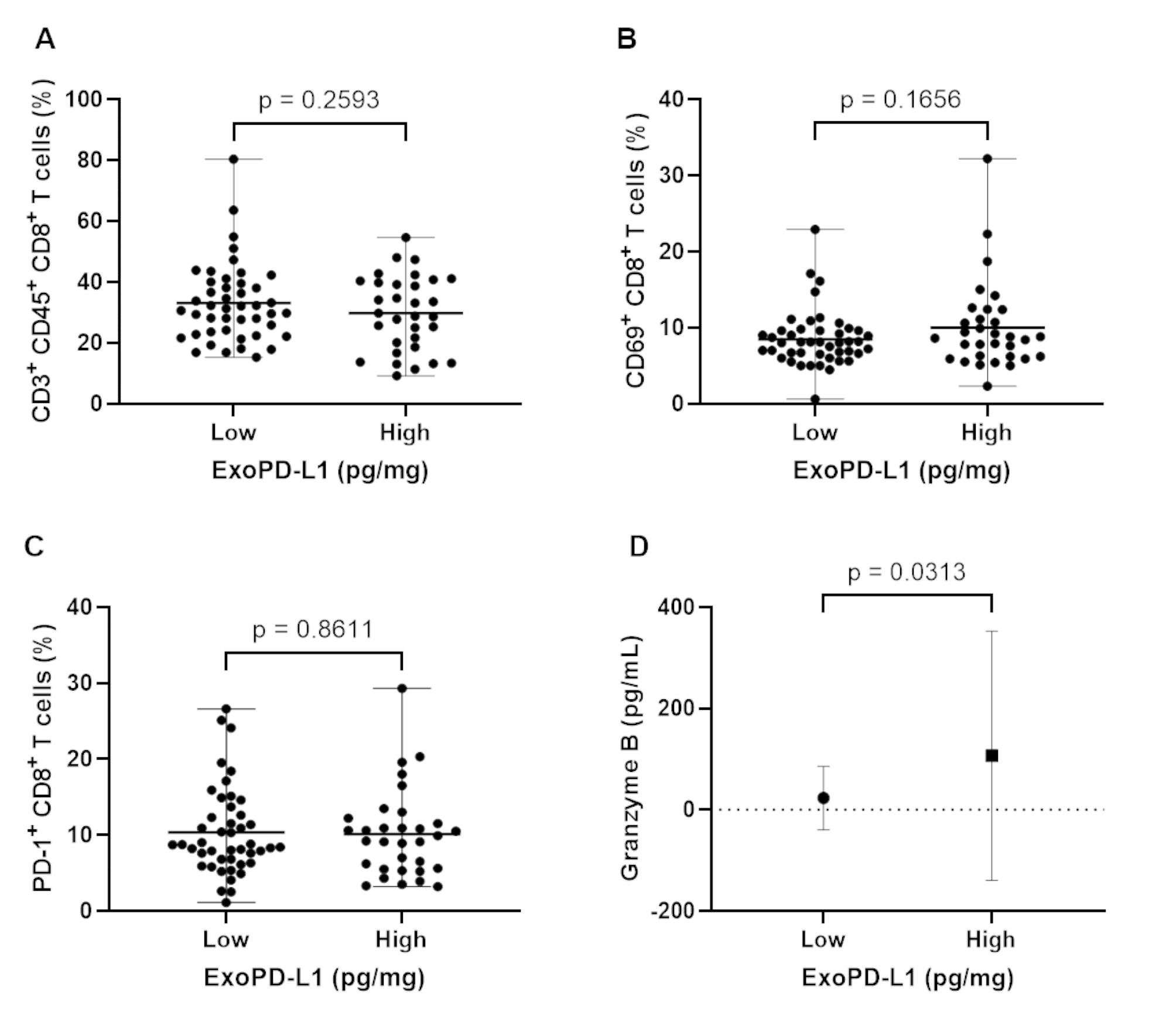
The proportion of cytotoxic T lymphocytes and concentration of granzyme B according to serum exoPD-L1 levels. No differences were observed between the two groups with respect to (**A**) total cytotoxic CD8 + T lymphocytes, (**B**) activated CD8 + T lymphocytes with CD69 expression, and (**C**) CD8 + T lymphocytes with PD-1 expression; however, (**D**) granzyme B concentration was higher in the high exoPD-L1 group

The proportion of CD3 + CD45 + CD8 + T cells was measured in each patient, and the fraction of CD69 + CD8 + T cells was assessed to investigate cytotoxic T cell activity. The total cytotoxic T cell (*p* = 0.259) and effector CD8 + T cell (*p* = 0.166) proportions were comparable between the two groups according to the exoPD-L1 level (Fig. 4A and B). The proportion of PD-1-expressing CD8 + T cells, which may interact with PD-L1-expressing tumors or exosomes, did not differ between the two groups (*p* = 0.861; Fig. 4C). Furthermore, no significant differences were observed in the serum levels of TGF-β1, TGF-β2, IFN-γ, and IL-1β between the two groups. However, the levels of granzyme B produced by CD8 + T cells to mediate cytotoxic activity were significantly higher in the high exoPD-L1 group than in the low exoPD-L1 group (*p* = 0.031; Fig. 4D).

## Discussion

Activation of the PD-1/PD-L1 signaling pathway can suppress T cell responses in various cancers, thereby leading to immune evasion [24]. To the best of our knowledge, this is the first study to investigate the clinical implications of exoPD-L1 expression with regard to prognosis in patients with advanced pancreatic cancer.

In a recent meta-analysis, elevated tumor PD-L1 expression was correlated with advanced stage and poor OS in patients with PDAC. However, the IHC methods for PD-L1 expression were not validated [11]. High plasma sPD-L1 is an adverse prognostic factor in gastric cancer and hepatocellular carcinoma, but not in pancreatic cancer [21, 22]. Moreover, although elevated sPD-L1 levels were negatively correlated with overall survival (OS) in advanced pancreatic cancer, this observation was limited by a small sample size [17]. On the other hand, positive expression of exoPD-L1 has been found to be associated with shorter postoperative survival in patients with pancreatic cancer [25]. In our study, patients with high levels of exoPD-L1 demonstrated numerically poorer median OS, although in a multivariate analysis, accounting for established prognostic factors, exoPD-L1 levels did not show a significant association with survival outcomes. These results suggest that PD-L1 blockade monotherapy may not be efficient in patients with advanced PDAC.

In this study, exoPD-L1 levels were not significantly correlated with clinicopathological features, except for the disease status. Patients with systemic disease had elevated exoPD-L1 levels compared to those with locally advanced disease, which was consistent with previous reports wherein exoPD-L1 levels correlated with advanced disease stage in patients with head and neck and lung cancer [15, 26]. The PD-L1-containing exosomes secreted by cancer cells are released into the lymph nodes and suppress anti-tumor T-cell activity. Furthermore, PD-L1-expressing exosomes facilitate immune escape of tumor cells and contribute to the formation of pre-metastatic niches [27]. Thus, blocking the secretion of PD-L1-containing exosomes from cancer cells could suppress disseminated metastasis.

We assessed the correlation between tumor PD-L1 IHC status and serum exoPD-L1 levels in patients with pancreatic cancer for the first time. Serum exoPD-L1 levels were higher in the positive than in the negative PD-L1 expression group. Consistently, in a previous study, tumor PD-L1 expression was associated with a higher percentage of PD-L1-expressing exosomes in patients with non-small cell lung cancer [28]. In contrast, sPD-L1 was not correlated with tumor PD-L1 expression in several types of cancers, including pancreatic cancer [22, 29–31]. Although exosomes could be secreted by immune cells apart from tumor cells, given that exoPD-L1 levels were associated with tumor PD-L1 expression in PDAC, a significant portion of serum PD-L1 expressing exosomes may have been secreted from tumor cells.

Similar to sPD-L1, serum-derived exoPD-L1 may be elevated in patients with increased systemic inflammation provoked by the inflammatory tumor type of pancreatic cancer [22]. Pancreatic cancer is considered non-immunogenic cancer with either poor infiltration of immune cells or considerable infiltration of immunosuppressive T cells [7]. However, as pancreatic cancer with increased sPD-L1, including exoPD-L1, exhibits strong tumor T-cell infiltration, a combination of anti-PD-1/PD-L1 therapy with immunomodulating agents that inhibit immunosuppressive T cells, such as Tregs, may have clinical benefits. In a recent study, the combined blockade of PD-L1 and CCL-5, which recruits Treg cells, strengthened antitumor effects in a xenograft model of pancreatic cancer [32]. To overcome the resistance of PD-1/PD-L1 targeted immunotherapy in pancreatic cancer, further studies are required to modulate the TME consisting of immunosuppressive cells such as Tregs, MDSCs, and TAMs.

Circulating exosomes can interact with immune cells through various mechanisms. PD-L1-containing exosomes can suppress CD8 + T-cell activity by downregulating CD69 expression on the surface of activated T cells [15]. Additionally, granzyme B is usually produced and secreted by CD8 + T cells as well as by natural killer and non-cytotoxic cells, such as basophils and mast cells [33]. However, we did not observe a significant association between exoPD-L1 levels and the proportion of effector CD8 + T cells. Contrary to expectations, granzyme B levels were higher in the high exoPD-L1 group than in the low exoPD-L1 group, probably because granzyme B could be induced by systemic inflammation. Prior research has not found significant associations between TGF-β and IFN-γ levels and prognosis in advanced pancreatic cancer patients, a result which was also observed in this study [34, 35]. While it was previously suggested that increased exoPD-L1 levels may be linked to reduced IFN-γ secretion from T cells [16], we did not find any significant differences between the high and low exoPD-L1 groups. IL-1β has been associated with immune suppression and inhibition of CD8 + T cell activation in pancreatic cancer [36], but our study did not observe a significant prognostic association with IL-1β.

Several limitations of our study need to be considered. First, the association between treatment resistance and exoPD-L1 was not evaluated because exoPD-L1 was not serially measured after treatment. Second, none of the patients in this study received anti-PD-1/PD-L1 blockade immunotherapy; therefore, we could not investigate the impact of exoPD-L1 on the response to anti-PD-1/PD-L1 blocking therapy. Third, tissue evaluation in relapsed patients was based on surgically resected specimens rather than relapsed tissues. Finally, the relationship between exoPD-L1 and tumor-infiltrating CD8 + T cells in the TME could not be investigated, as only serum-derived exosomes were evaluated and not exosomes from the tumor tissue.

In conclusion, we found that exoPD-L1 was higher in PDAC patients with systemic disease than in those with locally advanced disease. Serum-derived exoPD-L1 levels correlated with PD-L1 expression in tumor tissue. Additionally, patients with PDAC and high exoPD-L1 levels had worse survival outcomes than those with low exoPD-L1 levels. Further studies exploring the association between PD-L1 expressing exosomes derived from tumor tissue and cytotoxic or immunosuppressive T cells in the TME are required to validate the clinical importance of exoPD-L1 in patients with pancreatic cancer.

## Electronic supplementary material

Below is the link to the electronic supplementary material.

Supplementary Material 1

## Data Availability

The datasets used in the current study are available from the corresponding author on request.

## Abbreviations

PDAC: Pancreatic ductal adenocarcinoma
OS: Overall survival
TME: Tumor microenvironment
PD-L1: Programmed cell death ligand 1
PD-1: Programmed cell death receptor 1
ICI: Immune checkpoint inhibitors
Tregs: Regulatory T cells
MDSCs: Myeloid-derived suppressive cells
TAMs: Tumor-associated macrophages
sPD-L1: Soluble PD-L1
exoPD-L1: Exosomal PD-L1
ECOG: Eastern Cooperative Oncology Group
PS: Performance status
IRB: Institutional Review Board
ELISA: Enzyme-linked immunosorbent assay
TGF-β1: Transforming growth factor beta 1
IFN-γ: Interferon-gamma
IL: Interleukin
ROC: Receiver operating characteristic
IHC: Immunohistochemistry
CPS: Combined positive score
IQR: Interquartile ranges
HR: hazard ratio
CI: Confidence intervals
CA 19 − 9: Carbohydrate antigen 19 − 9
NLR: Neutrophil-to-lymphocyte ratio
