# Supplementary material for: Clinical significance of serum-derived exosomal PD-L1 expression in patients with advanced pancreatic cancer
Source: BMC Cancer. 2023 May 1;23:389. doi: 10.1186/s12885-023-10811-8 (PMC10150468; doi:10.1186/s12885-023-10811-8)
Supplement: Supplementary file 1 — Supplementary Material 1 [file 12885_2023_10811_MOESM1_ESM.docx]

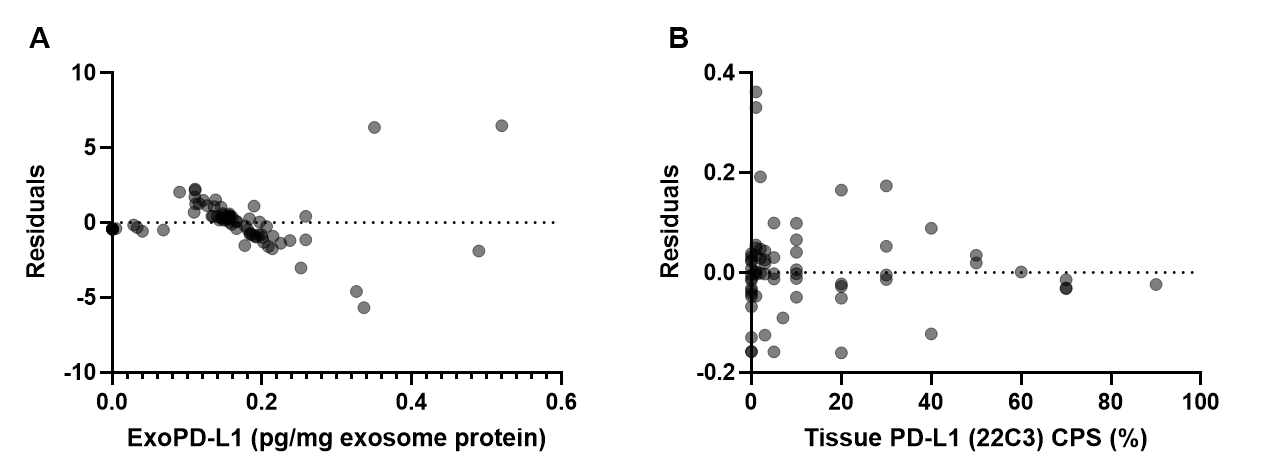


**Fig. S1 Residual plot.** (A) Simple linear regression of correlation between serum and relative exoPD-L1 concentrations. (B) Simple linear regression of correlation between tumor PD-L1 expression and serum exoPD-L1 concentrations.


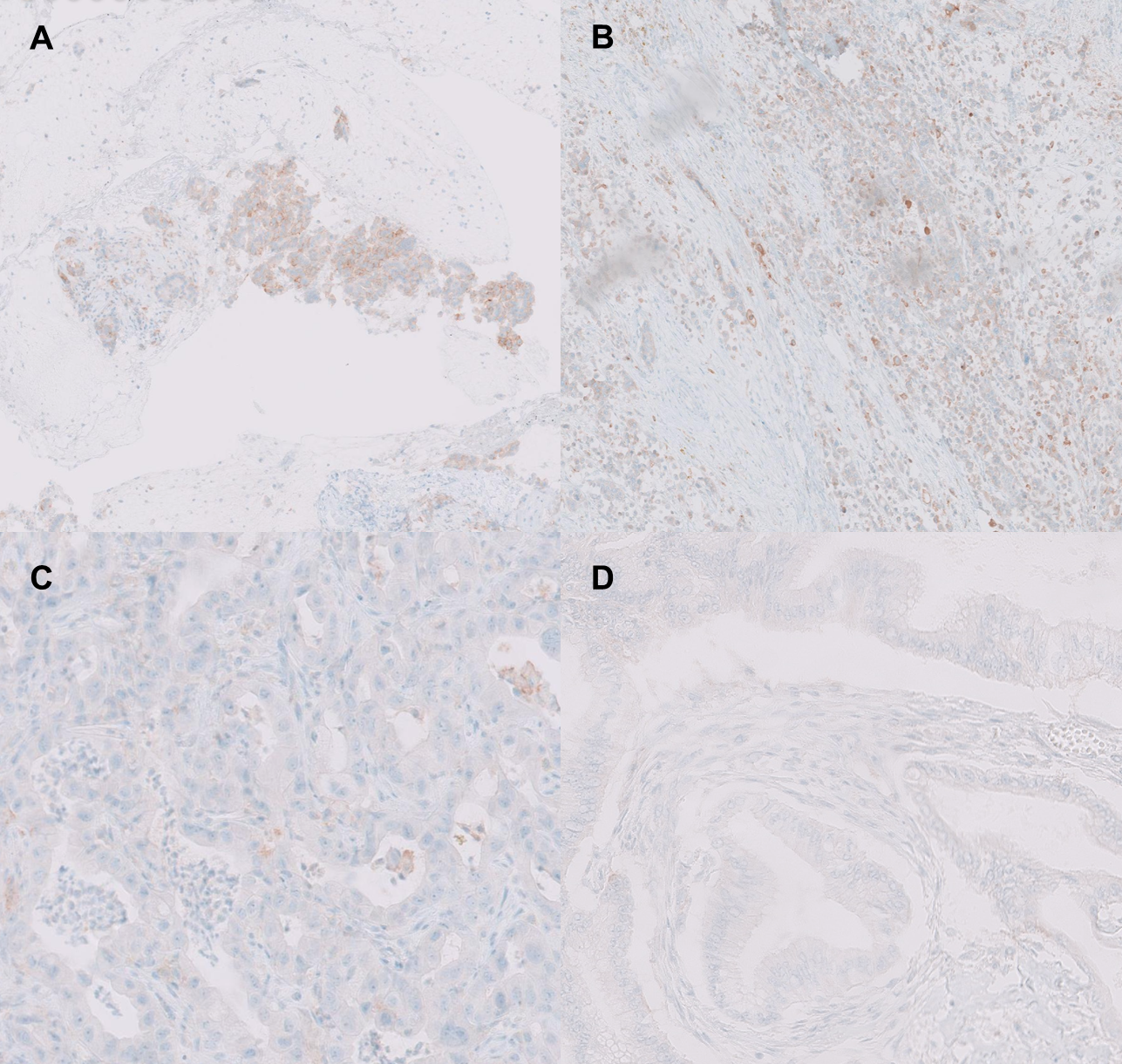


**Fig. S2 Immunohistochemical staining of PD-L1 using 22C3 pharmDx assay.** Tumor tissue PD-L1 positivity was defined as combined positive score (CPS) ≥ 1%. (A) CPS ≥ 50%, (B) 50%> CPS ≥ 10%, (C) 10%> CPS ≥ 1% and (D) CPS < 1% (PD-L1 negative expression).


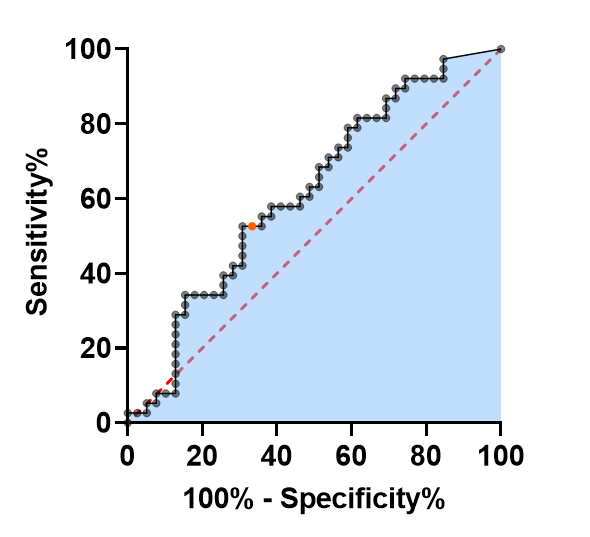


**Fig. S3 Receiver operating characteristic (ROC) curve analysis.** ROC curve analysis to determine the ideal prognostic threshold for exoPD-L1 in predicting survival.

**
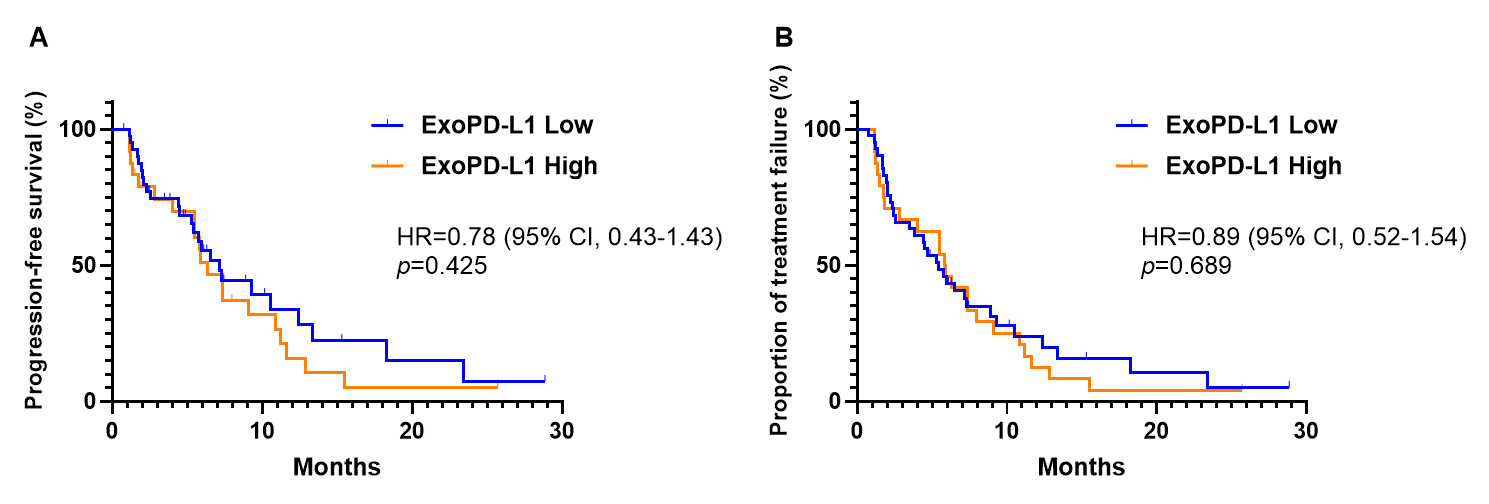
**

**Fig. S4 Kaplan–Meier estimates of progression-free survival and time to treatment failure according to serum exoPD-L1 levels.** (A) Progression-free survival and (B) time to treatment failure in patients who received systemic chemotherapy (n=65).

**
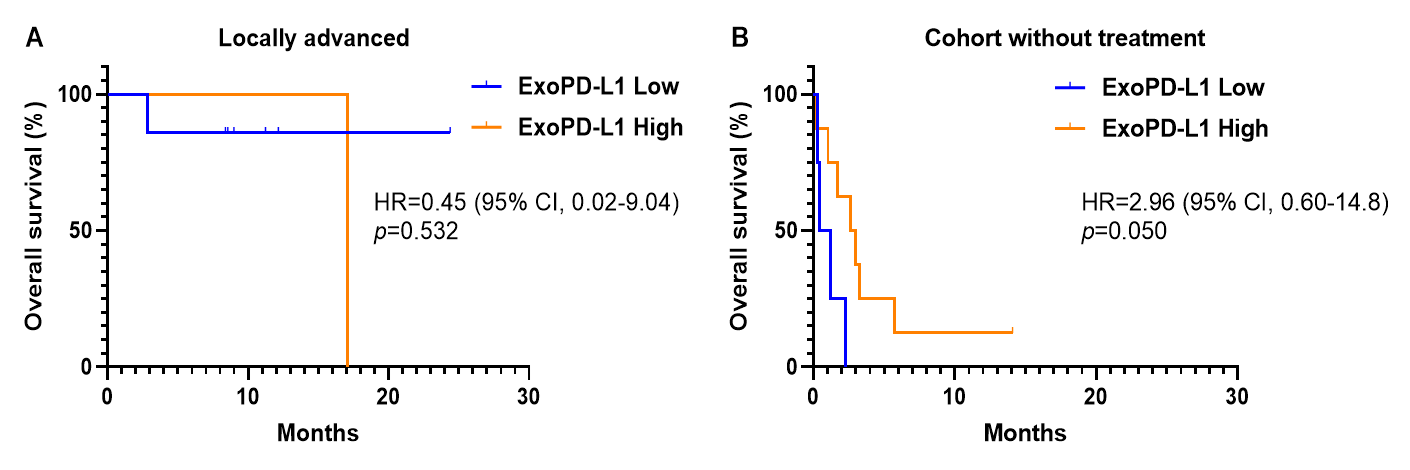
**

**Fig. S5 Kaplan–Meier estimates of overall survival according to serum exoPD-L1 levels.** (A) Overall survival in patients with locally advanced disease (n=8), and (B) in patients who did not receive systemic treatment (n=12).

**Table S1. Efficacy of systemic treatment according to serum exoPD-L1 levels**

| **Variables** | **Low exoPD-L1**  **(n=41)** | **High exoPD-L1**  **(n=24)** | ***p* value** |
| --- | --- | --- | --- |
| **Best response, n (%)**  Complete response  Partial response  Stable disease  Progressive disease | 0  5 (12.2)  21 (51.2)  15 (36.6) | 0  6 (25.0)  11 (45.8)  7 (29.2) | 0.406 |
| **Objective response rate, n (%)** | 5 (12.2) | 6 (25.0) | 0.184 |
| **Disease control rate, n (%)** | 26 (63.4) | 17 (70.8) | 0.542 |
| **Median PFS, months [95% CI]** | 10.0 [6.83-13.2] | 7.8 [5.26-6.52] | 0.425 |

*ExoPD-L1* exosomal programmed cell death ligand 1, *PFS* progression-free survival.
